# Infrared Thermal Imaging and Morpho-Physiological Indices Used for Wheat Genotypes Screening under Drought and Heat Stress

**DOI:** 10.3390/plants11233269

**Published:** 2022-11-28

**Authors:** Waseem Ashfaq, Graham Brodie, Sigfredo Fuentes, Dorin Gupta

**Affiliations:** 1 School of Agriculture and Food, Faculty of Veterinary and Agricultural Sciences, The University of Melbourne, Melbourne, VIC 3010, Australia; 2 College of Science and Engineering, James Cook University, Townsville, QLD 4811, Australia

**Keywords:** *Triticum aestivum* L., abiotic stress, climate change, early grain-filling, water stress, flag leaf senescence, high-temperature

## Abstract

Bread wheat, one of the largest broadacre crops, often experiences various environmental stresses during critical growth stages. Terminal drought and heat stress are the primary causes of wheat yield reduction worldwide. This study aimed to determine the drought and heat stress tolerance level of a group of 46 diverse wheat genotypes procured from the Australian Grains Gene Bank, Horsham, VIC Australia. Two separate drought stress (DS) and heat stress (HS) pot experiments were conducted in separate growth chambers. Ten days after complete anthesis, drought (40 ± 3% field capacity for 14 days) and heat stress (36/22 °C for three consecutive days) were induced. A significant genotype × environment interaction was observed and explained by various morpho-physiological traits, including rapid, non-destructive infrared thermal imaging for computational water stress indices. Except for a spike length in DS and harvest index in HS, the analysis of variance showed significant differences for all the recorded traits. Results showed grains per spike, grains weight per spike, spike fertility, delayed flag leaf senescence, and cooler canopy temperature were positively associated with grain yield under DS and HS. The flag leaf senescence and chlorophyll fluorescence were used to measure each genotype’s stay-green phenotype and photosystem II activity after DS and HS. This study identified the top ten best and five lowest-performing genotypes from drought and heat stress experiments based on their overall performance. Results suggest that if heat or drought adaptive traits are brought together in a single genotype, grain yield can be improved further, particularly in a rainfed cropping environment.

## 1. Introduction

Being the staple food of over 35% of the world’s population [1], bread wheat is the world’s most important grain crop, cultivated on almost 214 million hectares globally. With a total production of 765.8 million tonnes in 2019 [2], wheat provides around 20% of the total consumed vegetal proteins and calories to the 4.5 billion people worldwide [3]. In a Mediterranean region, wheat grain yield is increasingly affected by recurring climatic variations during anthesis and grain filling growth stages, raising concerns for adequate food supply in the future [4,5]. Previously, it was predicted that wheat yield should increase at the rate of 60% to meet world demand, but production targets might go down by 29% due to increased environmental stresses [6]. These predictions have demanded the development of wheat varieties better adapted to the changing climatic conditions with relevant agronomic and adaptive traits to improve biomass and grain yield, which is crucial for future agricultural productivity [7].

Among various environmental constraints, drought and heat stresses are two main factors that severely restrain crop growth and productivity worldwide [8]. Estimated ~40% annual production variability in major wheat-producing belts is mainly due to unpredictable drought and heat stress events [9]. Moderate to severe effects of these stresses drastically alter wheat’s morpho-physiological traits. Exposure to drought stress generally results in a significant reduction in the growth and yield of wheat [8]. A plant’s response to drought stress shows multiple morphological and physiological changes, which become more severe at later growth stages with functional damage to plant parts [10]. Some drought-induced effects include a reduced mass flow of water-soluble nutrients, higher canopy temperature due to closed stomata, infertile spikelets, reduced grain weight due to decreased carbohydrate supply and early senescence with shorter grain filling duration [8,11]. Plants use numerous adaptive traits to prevent injury in a dehydrating environment [12]. Early plant vigour improves nutrient uptake and weed competitiveness, which can enhance resilience to the erratic drought periods [13].

Wheat is highly sensitive to heat stress during critical phenophases [14]. The crop response to elevated temperature is influenced by various edaphic factors, especially evapotranspiration, growing season, and soil water content. It is also affected by synoptic events like the number and duration of hot days, maximum daily temperature, and critical developmental stages of the crop related to heat stress susceptibility [15]. The temperature threshold for grain filling ranges between 12–22 °C [8]. Above-optimal exposure during grain filling can lead to terminal heat stress and can result in a significant reduction in crop productivity. Some previously reported heat stress-induced effects include disintegration of the membrane structure, shortened crop life cycle, increased flag leaf senescence, reduced photosynthetic efficiency, higher canopy temperature, disturbed source-sink relationship, and adverse effects on spikelets formation during meiosis causing kernel abortion [16,17].

Drought and heat stress accelerates maturity and reduces leaf colour and chlorophyll contents, leading to premature plant senescence [18]. Photosystem II is highly sensitive to elevated temperature stress and may cause inevitable injury to the photosynthetic and respirational processes [19]. Assessing the photosystem II (PSII) stability through chlorophyll fluorescence analysis can form an effective, non-invasive, and reliable technique for a drought and heat stress study. Asseng et al. [20] conducted a modelling study in the Australian wheat belt to investigate the effect of elevated temperatures on Australian wheat varieties. They reported that an average 2 °C increase in growing temperature could reduce crop productivity by up to 50%, most of which can be associated with accelerated leaf senescence due to higher temperatures. Previously, thermal imaging was accurately used as an integrative approach to estimate crop water stress indices (canopy temperature (T_c_), crop water stress index (CWSI), and canopy temperature depression (CTD)) for drought and heat stress study in wheat. The T_c_ and CWSI fluctuate with the surrounding environment and negatively correlate with the final grain yield [21].

The screening of diverse germplasm is critical in identifying drought or heat-stress-tolerant and susceptible genotypes. This approach requires the utilization of appropriate phenotyping methods, identification of the accurate developmental stage most prone to stress events, and relevant traits of interest for a stress tolerance study [22]. Based on that, the identification and adoption of tolerant genotypes with desirable adaptive traits are the most sustainable ways to safeguard crop productivity in a changing climate. Selecting for such improved attributes has significantly enhanced crop productivity in rainfed conditions [23]. Despite the fact that a lot of progress has been made in drought and heat stress tolerance studies in wheat, to the best of our knowledge, there is currently limited published data available quantifying various genotypic performances based on their morpho-physiological attributes, including non-destructive computational water stress indices in a terminal drought and heat stress environments. Thus, in the current study, a group of 46 wheat genotypes, primarily Australian, were exposed to heat and drought stress during their early grain-filling stages. The study aim includes (1) determining if there exists genetic variability and stress tolerance efficiency within the adapted plant genetic resources for terminal heat and drought stress tolerance and (2) exploring various morpho-physiological traits, including infrared thermal imaging (IRTI) technique, as high-throughput phenotyping methodology for water stress indices at an early grain filling stage to facilitate the screening process to discriminate terminal drought or heat tolerant and susceptible genotypes.

## 2. Material and Methods

### 2.1. Plant Material, Experimental Design and Establishment

Forty-six wheat genotypes (G1–G46), including five checks (G1–G5), were procured from the Australian Grains Genebank, VIC Australia (Table 1). A non-replicated augmented randomized complete block design [24] was used to evaluate the test genotypes for drought and heat stress tolerance in two parallel drought and heat stress experiments, hereafter referred as E1 (drought screening experiment) and E2 (heat screening experiment). Tolerant (RAC875, Excalibur, Drysdale, Axe) and susceptible (Kukri) checks were used for comparison [25]. All 41 test genotypes (G6–G46) were grouped into four blocks (*b*_1_ = G6–G15, *b*_2_ = G16–G25, *b*_3_ = G26–G35, *b*_4_ = G36–G46). The checks were replicated in each of the four blocks, while test genotypes were grown only once in the experiment. The repetition of checks enables the estimation of the error variance and blocking effects for statistical inference, resulting in more precise estimates of the treatment comparison of interest. The position of the test genotypes and the checks were fully randomized in each block.

The genotypes were sown in polypropylene square-shaped nursery pots (9.5 × 7 cm, height × diameter) with five biological replicates in plant growth chambers (PGR15) at the Dookie Campus, The University of Melbourne, Australia (−36.384° S, 145.707° E) from October 2018 to February 2019. Initially, eight seeds of each genotype were sown per pot and later thinned to five after ten days of sowing for optimum plant growth. The three harvested seedlings were used to destructively measure the early vigour of wheat genotypes.

For the experiment, the soil was collected from the upper 15–20 cm layer of a cultivated field from the mount major lane wheat paddock of Dookie Campus and sieved well with 4 mm mesh to remove plant debris and stones for homogenization. Each pot was filled with 6 kg of sieved soil. The soil was red-brown Dookie clay loam, classified as Gowangardie loam [26]. The soil analysis report showed no issues with toxicities or mineral deficiencies associated with the soil. Specifically, the soil contained 2.1% organic carbon, 77 mg kg^−1^ available N, 210 mg kg^−1^ Colwell-P, and 660 mg kg^−1^ available K. Standard agronomic practices and appropriate fertilization were implemented to minimize yield limitations.

### 2.2. Growing Conditions

For ideal growing conditions, the PGR15 were programmed at 24 ± 1 °C and 18 ± 1 °C (13/11 h) for the day and night period [11]. Plant growth chambers were equipped with high-pressure sodium lamps for red, blue, and far-red light spectrums with high-intensity discharge using metal halide. The light intensity was adjusted to 450–500 μmoles m^−2^ s^−1^ above the plant canopy. The relative humidity (RH) of the PGR15 was set to 65–70% [27].

### 2.3. Treatment Application

**Figure 1:**
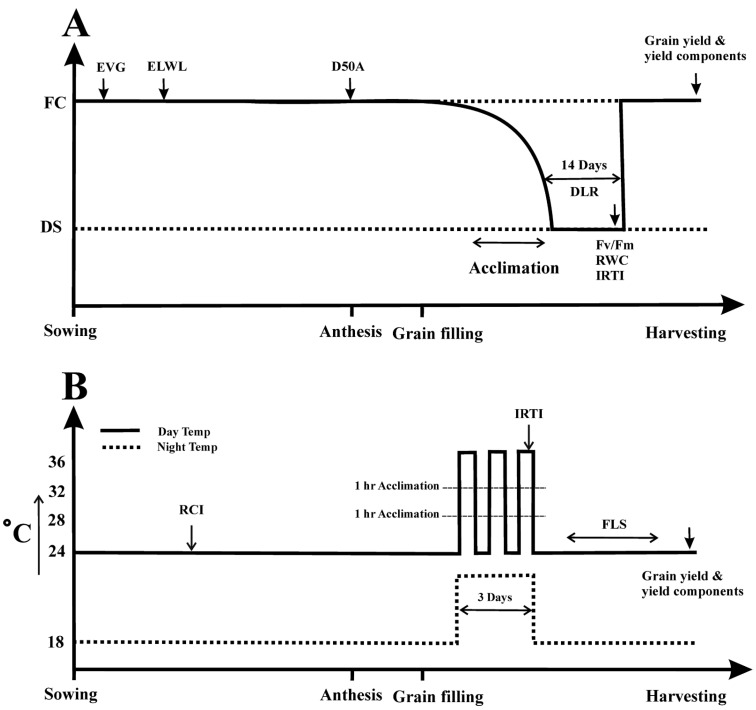
Schematic diagram of (**A**) terminal drought and (**B**) heat stress application with the time of traits measurement during the experiments. Abbreviations are (**A**) drought stress (DS); field capacity (FC); early vigour (EVG); excised leaf water loss (ELWL); days to 50% anthesis (D50A); chlorophyll fluorescence (F_v_/F_m_); relative water contents (RWC); infrared thermal imaging (IRTI). (**B**) relative cell membrane injury (RCI); flag leaf senescence (FLS).

The genotypes were individually monitored for their anthesis time and split into three groups according to the early, mid, and late maturity life cycle. Before drought or heat stress treatment, each group was monitored to determine when their primary tiller reached the first phase of grain filling, ten days after complete anthesis (Zadoks growth stage 75) [28,29]. At this stage, the separate extreme event of drought stress (DS) and heat stress (HS) were imposed on the designated pots of E1 and E2. For the HS treatment, designated pots from E2 were subjected to high-temperature acclimation for three whole days with day/night temperatures of 36/22 °C for 8 and 12 h, respectively [29,30]. Before and after eight hours of heat stress (36 °C), the PGR15 were programmed in a periodic pattern for gradual increase and decrease in the day and night temperature (Figure 1). The RH was set at 50% during the HS. Irrigation was normally applied to remove any confounding effects of drought on heat-stressed plants. After the three days of HS, the optimum growing temperature was programmed again in the PGR15 for the remaining plant life cycle.

For DS treatment, water stress with 40 ± 3% pot field capacity (FC) was imposed on E1 pots for an entire 14-days period. Before the start of the DS, irrigation was slowly reduced for five days to attain 40 ± 3% FC (Figure 1). The RH was set to 60% during the DS period. From the start of the DS, pots were weighed daily to measure the amount of water loss by evapotranspiration. Based on that, pots were irrigated accordingly to maintain the FC at the required level. After 14 days of DS, regular irrigation was resumed, and full FC of the experiment (pots) was kept for the remaining plant’s life cycle. The development of DS was monitored in destructive (estimation of relative water contents; RWC) and non-destructive ways (assessment of chlorophyll fluorescence with Mini-Palm-II). During both experiments, the pots were rotated inside the growth chamber after every 2nd week to minimize any favouring effects.

### 2.4. Data Collection

#### 2.4.1. Recorded Parameters for Drought Stress Experiment

##### Early Vigour Estimation

Following Rebetzke and Richards [31] method, the early vigour (EVG) of each genotype was recorded with slight modification. The measurement of EVG components consisted of the 1st, 2nd, and 3rd leaf area of the individual plant along with their phyllochron interval (the thermal time interval between two successive leaf tips). The phyllochron interval of the first leaf was calculated by computing growing degree days from the day of sowing to the emergence of the first leaf. Phyllochron interval of the second and third leaf consisted of growing degree days from sowing to the emergence of the second and third leaf, respectively. The leaves were excised just before the emergence of the 4th leaf to measure the leaf area. The sum of the area of the first three leaves with respective phyllochron intervals was used as the measure of EVG.

##### Excised Leaf Water Loss

The excised leaf water loss (ELWL) of each genotype was estimated using the CLARKE McCAIG [32] method. Fully developed young leaves of 1-month-old plants were excised and weighed instantly for the fresh weight (FW_0_). After the initial weight, the leaves were immediately shifted to a controlled growth room maintained at 20 °C with 50–60% of RH and 250–300 µmol m^−2^ s^−1^ of continuous light. The leaf weights were recorded after four hours (FW_4_), eight hours (FW_8_), and finally after drying (DW) at 65 °C for 48 h. The excised leaf water loss after four hours, eight hours, and from four to eight hours was estimated as a percentage of water loss per unit of initial water contents as follows.

(1) $${\mathrm{ELWL}}_{0–4h\;}\left(\%\right)=\left[\left({\mathrm{FW}}_{0}-{\mathrm{FW}}_{4}\right)/\left({\mathrm{FW}}_{0}-\mathrm{DW}\right)\right]\times 100$$

(2) $${\mathrm{ELWL}}_{4–8h\;}\left(\%\right)=\left[\left({\mathrm{FW}}_{4}-{\mathrm{FW}}_{8}\right)/\left({\mathrm{FW}}_{4}-\mathrm{DW}\right)\right]\times 100$$

(3) $${\mathrm{ELWL}}_{0–8h\;}\left(\%\right)=\left[\left({\mathrm{FW}}_{0}-{\mathrm{FW}}_{8}\right)/\left({\mathrm{FW}}_{0}-\mathrm{DW}\right)\right]\times 100$$

##### Relative Water Contents

The sections of leaves (8–10 cm long) were taken on the last day of the DS treatment. The fresh weight of leaf samples (FW) was determined instantly after excision, followed by soaking in distilled water in plastic tubes for 12 h at standard room conditions. The hydrated samples were then taken out of the distilled water, gently dried with tissue paper, and reweighed for the turgid weight (TW). The leaf samples were then oven-dried at 70 °C for 48 h for their dry weight (DW). Following Barrs and Weatherley [33], the RWC of each genotype was calculated using the following formula.

(4) RWC (%)=[(FW−DW)/(TW−DW)]×100

##### Chlorophyll Fluorescence

Before the end of the DS treatment, the chlorophyll fluorescence (F_v_/F_m_) of each genotype was measured with a portable LED modulation fluorescence system (Mini-Palm-II, Heinz Walz, Germany). Minimal chlorophyll fluorescence (F_o_) was determined in dark-adapted leave discs (30 min) under low modulated actinic light (>0.04 μmolm^−2^ s^−1^) followed by a 1-s saturated white light pulse (>3000 μmolm^−2^ s^−1^) in the discs of same leave to estimate maximal chlorophyll fluorescence (F_m_). The quantum yield of photosystem II for dark-adapted leaves was calculated as reported by Carvalho and Amâncio [34].

(5) $$F_{V}/F_{m}=\left(F_{m}-F_{o}\right)/F_{m}$$

#### 2.4.2. Recorded Parameters for Heat Stress Experiment

##### Thermostability of Cell Membrane

The thermostability of the cell membrane was evaluated by following the Martineau et al. [35] method. A set of one cm squared-shaped leaf discs were made from the leaves of one-month old seedlings, placed into two test tubes, and rinsed thoroughly with distilled water to remove any surface bond electrolytes. After washing, the test tubes were filled with 10 mL of distilled water to immerse the washed leaf discs. One of the two test tubes was warmed in a water bath at 45 °C for an hour. After that, each test tube was incubated in the lab incubator in darkness at 10 °C for 24 h to allow the diffusion of free electrolytes from the leaf discs. The next day, after shaking well, the control and treated samples (C_1_ and T_1_, respectively) were recorded with the TPS AQUA-CPA Cond/Salinity meter. After the initial readings, test tubes were put together for a 20 min autoclave at 120 °C and 100 kPa for a second conductance reading (C_2_ and T_2_, respectively). The thermostability of the cell membrane was calculated as a relative cell membrane injury percentage (RCI, %) using the following formula.

(6) $$\mathrm{RCI}\;\left(\%\right)=\left[1-\frac{1-\left(T_{1}/T_{2}\right)}{1-\left(C_{1}/C_{2}\right)}\right]\times 100$$

##### Flag Leaf Senescence

The flag leaf senescence (FLS) of all studied genotypes was estimated, starting from one week after heat stress to physiological maturity. The loss of green leaf area (SPAD readings) after HS and during the recovering period in an index of the FLS. A Minolta SPAD-502 plus chlorophyll meter was used to measure the FLS of all five plants of each genotype. The measurements were carried out at weekly intervals for six weeks. Around 1 cm from the edges, three readings were taken downside of the leaf from the base, centre, and tip.

The relative rate of flag leaf senescence at physiological maturity (FLS_m_) was calculated by adapting ‘Equation (5)′ from the Borrell et al. [36] and expressed as a percentage loss of relative green leaf area per day.

(7) $${\mathrm{FLS}}_{m\;}\left(\%\right)=\frac{\left[\left(1-{\mathrm{SPAD}}_{m}/{\mathrm{SPAD}}_{a}\right)\times 100\right]}{\mathrm{Days}\;\mathrm{from}\;10\;\mathrm{days}\;\mathrm{after}\;\mathrm{anthesis}\;\mathrm{to}\;\mathrm{maturity}}$$

where SPAD_a_ and SPAD_m_ are SPAD readings taken after weekly intervals of heat stress and physiological maturity, respectively.

##### Infrared Thermal Imaging-Based Computational Water Stress Indices

Before the end of the drought and heat stress, infrared thermal images (IRTI) of each genotype were taken using a thermal camera (Model 1050sc, FLIR Systems AB, Täby, Sweden). At 30 °C, the spatial resolution and thermal sensitivity of the camera was 0.47 milliradians and <20 mK, respectively. The object temperature measurement range of the camera is −40 °C to 150 °C. Each Pixel of IRTI holds a 16-bit thermal value in a °C unit. The thermal images were taken in standard room conditions to avoid plant canopy temperature acclimatization with the surrounding environment. To calculate the canopy temperature (T_c_) and crop water stress index (CWSI) [37,38], the thermal images were analyzed using MATLAB code scripted in MATLAB R2017b (Math works Inc., Natick, MA, USA) and Images Analysis Toolbox [21,37,39] through .dat image format.

(8) $$\mathrm{CWSI}=\frac{\left(T_{c}-T_{\mathrm{wet}}\right)}{\left(T_{\mathrm{dry}}-T_{\mathrm{wet}}\right)}$$

where T_c_ is the plant canopy temperature (°C), T_wet_ is the reference temperature (°C) of a non-stressed plant, and T_dry_ is the reference temperature (°C) of the stressed plant. T_wet_ and T_dry_ are empirical reference temperatures estimated through the statistical frequency analysis of IRTI.

Using T_c_ and empirical reference temperature data, an Infrared index (I_g_) of crop stress indices, which is proportional to the stomatal conductance (g_s_) and water vapour transport (g_w_), was estimated using the relationship proposed by Jones et al. [40] and Fuentes et al. [37].

(9) $$I_{g}=\frac{T_{\mathrm{dry}}-T_{c}}{T_{c}-T_{\mathrm{wet}}}=g_{w}(r_{\mathrm{aw}}+(\frac{s}{\gamma })r_{\mathrm{HR}})$$

where r_aw_ is the resistance to g_w_ in the boundary layer/air, “s” is the slop of the curve relating temperature with saturating vapour pressure, γ is the psychrometric constant, and r_HR_ is the parallel leaf resistance to the heat transport and radiative heat loss.

Canopy temperature depression (CTD) was also determined from IRTI analysis and calculated by subtracting the T_c_ from mean environmental temperature (T_a_) and it is positive when the plant canopy is cooler than the surrounding environment.

(10) $$\mathrm{CTD}=T_{a}-T_{c}$$

### 2.5. Agronomic Traits

The agronomic traits, grain yield, and yield components were estimated for both experiments. The days to 50% anthesis (D50A) was recorded as the total day count from sowing to extrusion of anthers in more than 50% spikes (Zadok stage 69) [28]. Plant height (PHT) was measured before two weeks of physiological maturity as the stretch from the ground to the spike tip, excluding awns. The grain yield (GY) of each genotype was determined using a standard protocol as summarized by Gahlaut et al. [41]. The grain weight per spike (GWS) and grains number per spike (GNS) were calculated as the average grain weight (gm) and average grain number of five spikes per genotype, respectively. The harvest index (HI) was measured as the ratio of GY to the total above-ground dry matter. The spike fertility (SF) was computed by dividing the grain number to the total number of spikelets of a spike in each genotype. Spike length (SL), spike number per plant (SN), and spikelets per spike (SPS) of all five plants were also calculated. For the DS experiment, the days to leaf rolling (DLR) of each genotype was also estimated by counting from the start of DS to the day when all leaves became rolled [42].

### 2.6. Statistical Analysis

An analysis of variance (ANOVA) was performed separately for both experiments using the “augmentedRCBD” package of R statistical software [43,44]. For the yield components, a descriptive variability analysis such as phenotypic variance (δ^2^p), genotypic variance (δ^2^g) and environmental variance (δ^2^e), phenotypic, genotypic, and environmental coefficient of variance (GCV, PCV and ECV, respectively), category of genotypic and phenotypic coefficient of variance, genetic advance (GA), and genetic advance as percent over mean (GAM) were computed from an object of class of “augmentedRCBD” package.

The thermal images data was extracted using MATLAB code scripted in MATLAB R2017b [39] and Images Analysis Toolbox for T_c_, CTD, I_g_, T_dry_, T_wet_, and CWSI [37] and analyzed using R statistical software. A principle component analysis was performed separately for both drought and heat stress experiments using “FactoMiner” and “factoextra” packages of R software to show the placement of genotypes on the factor map based on recorded morpho-physiological traits [45,46]. The correlation matrix for both E1, E2, and other presented graphs were made using a “ggplot2” package of the R software [47].

## 3. Results

### 3.1. Genotypic Differences for Early Growth and Development

**Figure 2:**
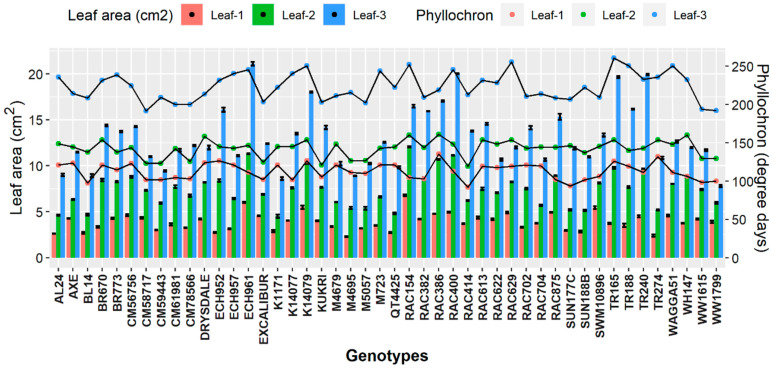
Early vigor components (area of leaf-1, leaf-2 and leaf-3 with respective phyllochron intervals) of 46 wheat genotypes. All values are presented with a mean of leaf area-1, 2 and 3 of three biological replicates with ±standard error (SE), SE represented with error bars.

The genotypic effects for EVG were significant (*p* < 0.05) for all the wheat genotypes and revealed associations among EVG components (Figure 2). Genotypes (i.e., ECH961, RAC400, TR240 and TR165) have had longer and broader leaves but showed slower growth due to higher growing degree days units. Similarly, Drysdale, Excalibur, RAC875, CM59443, WW1799, and ECH957 had the shortest phyllochron interval due to lower growing degree days and better leaf area components.

### 3.2. Genotypic and Phenotypic Variability Analysis for Grain Yield and Yield Components

The analysis of the variance of yield-associated traits for E1 and E2 showed significant variation within the genotypes (*p* < 0.05). Except for spike length in DS and harvest index in HS, highly significant differences were observed for all the measured traits within the main effects of blocking on treatment and their interactions (Table 2).

**Figure 3:**
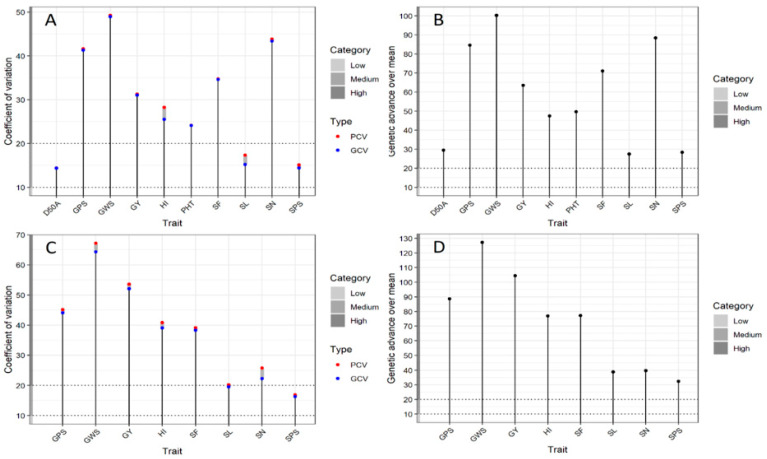
Phenotypic and genotypic coefficient of variability analysis for various agronomic traits of 46 wheat genotypes grown under drought (**A**,**B**) and heat-stressed (**C**,**D**) conditions. Abbreviations are: days to 50% anthesis (D50A); grains per spike (GPS); grains weight per spike (GWS); grains yield (GY); harvest index (HI); plant height (PHT); spike fertility (SF); spike length (SL); spike number per genotype (SN); spikelets per spike (SPS).

Similarly, Table 3 shows the significant impact of DS and HS on genetic variability within the genotypes. The phenotypic expression was strongly influenced by genotype × treatment interactions. The spike fertility exhibited maximum phenotypic and genotypic variance (PV and GV) under DS (381.9 and 378.9, respectively) and HS (450.8 and 431.8, respectively) followed by plant height (148.3 cm and 148.0 cm, respectively) in DS and harvest index (110.3 and 100.7, respectively) in HS. The phenotypic, genotypic, and environmental coefficient of variance (PCV, GCV and ECV) were also computed for the yield-related traits. The yield-components of all the genotypes had medium to high PCV and GCV in both experiments. The extent of treatment effects on measured traits is explained by the magnitude of variation among PCV and GCV. The maximum GCV was observed in grains weight per spike both in DS (48.9 gm) and HS (64.4 gm) followed by spike numbers in DS (43.3) and grain yield in HS (52.1 gm). It was observed the PCV was slightly higher than GCV for all the recorded traits. The smaller difference between PCV and GCV implies that recorded traits are less influenced by the growing environment for each genotype, exhibiting more genetic control (Table 3). Under DS and HS, grains per spike, grains weight per spike, spike fertility, and spike number falls within the high PCV and GCV categories (Figure 3A,C). Similarly, the genetic advance percent over mean (GAM) estimate ranged from 27.4 percent in spike length to 100.3 percent in grains weight per spike under DS compared to 32.4 percent in spikelets per spike to 127.2 percent in grains weight per spike under HS. Under DS and HS, grain yield and other yield-associated traits like grains per spike, grains weight per spike, spike fertility, spikelets per spike, spike length, and spike number fall within the high PCV and GCV categories. A lower GAM was observed in spike length and spikelets per spike both in DS and HS (Figure 3B,D).

### 3.3. Sensitivity of Wheat Genotypes to Drought and Heat Stress

Significant differences (*p* < 0.05) were observed across all the wheat genotypes for the mean values of recorded traits under DS and HS. Table 4 summarizes the adjusted mean values for the measured traits for the drought stress experiment and shows ten tolerant and five low-performing genotypes. The highest grain yield was recorded in ECH957 (8.87 gm), followed by M723 (5.97) and RAC875 (5.60 gm), while the lowest was recorded in CM59443 (2.55 gm) followed by TR274 (2.57 gm) under DS (Table 4). The early maturing genotypes were RAC704, WH147, and RAC875, taken 47.29, 47.32, and 47.73 D50A, respectively for the start of anthesis. Except for BR670 in the top-performing group, all other genotypes had cooler canopy temperatures than the surrounding environment. Genotypes like Drysdale, Excalibur, and RAC875 had the lowest CWSI under DS. Based on the visual score of leaf rolling, M723, BR670, ECH957, and RAC875 had the maximum days to leaf rolling duration, compared to low-performing genotypes (Kukri and TR274). The lowest ELWL among top-performing genotypes was observed in BR670 (45.77%), M723 (46.69%), and RAC875 (52.81%). Similarly, WH147 had the highest grains number per spike (28.47) among the group of top-performing genotypes, while ECH957 had the highest grains weight per spike (0.98 gm) followed by RAC875 (0.52 gm) and WH147 (0.51 gm), respectively. Among the low performing group, TR165 and Kukri were on the top for grains per spike (17.87 and 13.67, respectively). The tallest genotypes were ECH957 and M723, with mean PHT of 80.63 cm and 71.97 cm, respectively. RAC622 had a higher RWC (83.89%), followed by BR670 (81.19%) and RAC875 (78.77%). Drysdale, Excalibur, and RAC875 had a maximum I_g_, which indicates good stomatal conductance activity of these genotypes for keeping lower T_c_ (22.02 °C, 22.25 °C, 23.28 °C, respectively) under DS. Genotypes like WH147, Excalibur, RAC875, and ECH957 (top-performing genotypes) had the maximum spike fertility compared to CM59443 and TR274 (low-performing genotypes). Based on ranking criteria, the genotypes ECH957, RAC875, WH147, Drysdale, Excalibur, M723, RAC622, RAC704, AL24, and BR670 were considered drought-tolerant genotypes, while the genotypes Kukri, ECH952, TR165, TR274, and CM59443 were considered drought susceptible genotypes (Table 4).

Similarly, Table 5 summarizes the adjusted mean values for all the measured traits for E2 and shows the best-performing ten and low performing five genotypes under HS. The highest GY was observed in ECH957 (11.35 gm), followed by Axe (10.73 gm), TR188 (10.40 gm), Drysdale (10.03 gm), and RAC875 (9.90 gm), compared to Kukri (5.88 gm), CM59443 (2.74 gm), TR274 (1.90 gm), CM78566 (1.16 gm), and WH147 (1.16 gm) (low performing genotypes). The lowest percentage of relative cell membrane injury was recorded for RAC622 (5.58%), TR188 (10.68%), TR240 (11.41%), and Axe (12.16%) from the top-performing group. Compared to other genotypes, RAC875 and Excalibur had cooler canopy temperatures (25.20 °C and 25.66 °C, respectively) than the T_a_ and lower CWSI under HS. For yield-related components, genotypes like ECH957, RAC875, Axe, and Drysdale performed better than the rest of the genotypes in the top-performing group. For T_c_ and reference temperature (T_dry_ and T_wet_), ECH957, RAC875, RAC622, CM61981, WAGGA51, and Excalibur had minimum values under HS. Based on ranking criteria, genotypes ECH957, Axe, TR188, RAC875, RAC622, CM61981, WAGGA51, and Excalibur were considered heat-tolerant genotypes, while Kukri, CM59443, TR274, CM78566, and WH147 were considered heat susceptible wheat genotypes (Table 5).

### 3.4. Cumulative Genotypic Expression for Flag Leaf Senescence and Chlorophyll Fluorescence

**Figure 4:**
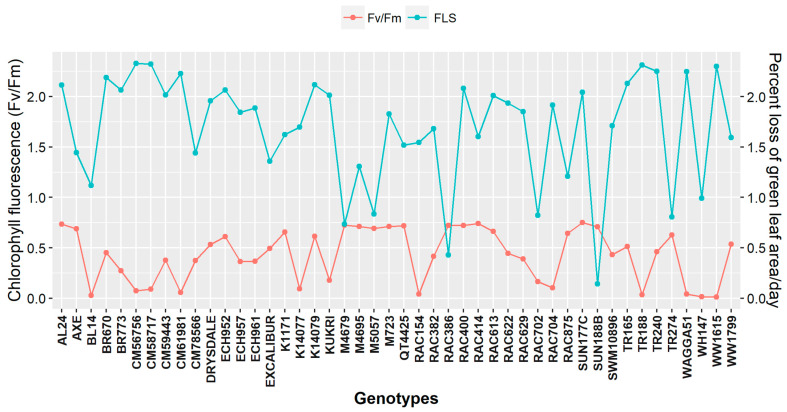
Non-destructive measurement of the relative rate of flag leaf senescence (FLS) as percent loss of the relative green leaf area per day for heat stress and chlorophyll fluorescence (F_v_/F_m_) for drought stress experiment.

A significant effect of HS (*p* < 0.05) was observed on the flag leaf chlorophyll content in all the genotypes (Figure 4). Heat stress accelerated the FLS with varying extents depending upon genotypic tolerance or susceptible behaviour. There was no significant difference in the FLS of the genotypes until the 3rd week after HS. A decline in the flag leaf greenness of all the genotypes was observed from the start of the 4th week after HS. At the end of the 5th week after HS, genotypes including BL14, SUN188B, RAC702, RAC875, RAC386, and TR274 recorded the lowest decrease in the percentage loss of green leaf area in a day.

For F_v_/F_m_, genotypes like Drysdale, ECH952, Excalibur, RAC875, and QT4425 had maximal quantum efficiency of PSII. Although DS inhibited F_v_/F_m_ across all genotypes, inhibition was more intense in BL14, RAC154, TR188, WAGGA51, WH147, and WW1615. Based on the cumulative result for both FLS and F_v_/F_m_, Excalibur, M4679, M5057, RAC386, RAC875, SUN188B, and TR274 were considered as stay-green genotypes with delayed leaf senescence. In contrast, genotypes like BL14, CM56756, CM58717, CM59443, CM61981, Kukri, RAC154, RAC704, TR188, WAGGA51, and WW1615 had faster leaf senescence following treatment effect and considered as susceptible genotypes.

### 3.5. Multivariate Data Analysis

**Figure 5:**
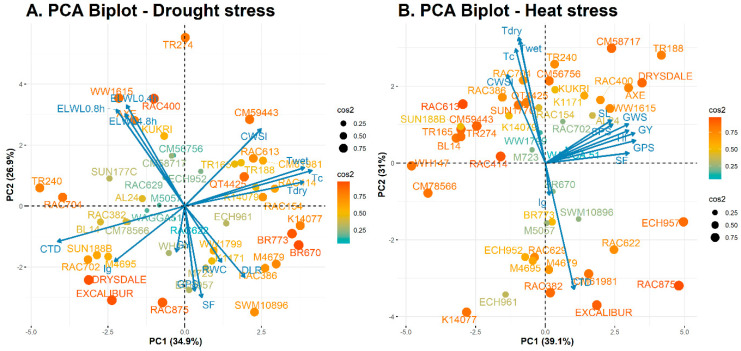
Principal component biplot showing genotype-by-trait interrelationship for (**A**) drought and (**B**) heat-stressed experiment. The magnitude of genotype-by-trait interrelationship represents by squared cosine (cos^2^) on the factor.

The principal component analysis (PCA) from the standardized genotypes-by-traits data matrix explained a total cumulative variance of 61.8% for the first two principal components (PC1 = 34.9% and PC2 = 26.9%) for the E1 and 70.1% (PC1 = 39.1% and PC2 = 31%) for the E2 (Figure 5A,B). Table 6 summarizes the rotated PC matrix for both E1 and E2, showing explained variance, proportion of total variance (%), and cumulative variance (%) by the first two PCs. Under drought, variables like T_dry_, T_wet_, and T_c_ had a higher positive loading in PC1. In contrast, variables like CWSI, and T_wet_ had a higher positive factor loading in PC2. Similarly, under heat stress, variables like grain yield, grains per spike, harvest index, spike fertility, spike length, grain weight per spike and spikelets per spike had a positive factor loading in PC1. In contrast, T_dry_, T_c_, and CWSI had a higher positive factor loading in PC2.

The relationship between variables and wheat genotypes concerning PCs under DS and HS are further elaborated through principal component biplot analysis (Figure 5). The PCA biplots were obtained from all the 46 wheat genotypes including higher factor loading parameters. The genotype excelling in a specific trait was plotted in the same direction and adjacent to the vector line of that particular trait vector. For example, under DS, the genotypes like ECH957, RAC875, and Excalibur excelling in the grain yield were mostly due to higher grains per spike and spike fertility. At the same time, the grain yield of these genotypes was negatively correlated with CWSI and T_c_ due to their obtuse angles with grain yield under DS (Figure 5A). Similarly, under HS, genotypes like QT4425, RAC704, and SUN177C had a higher T_c_, primarily due to higher CWSI, T_dry_, and T_wet_ compared to yield components (spike length, grains weight per spike, grain yield, grains per spike, spike fertility, and harvest index) due to their obtuse angle with T_c_ (Figure 5B).

**Figure 6:**
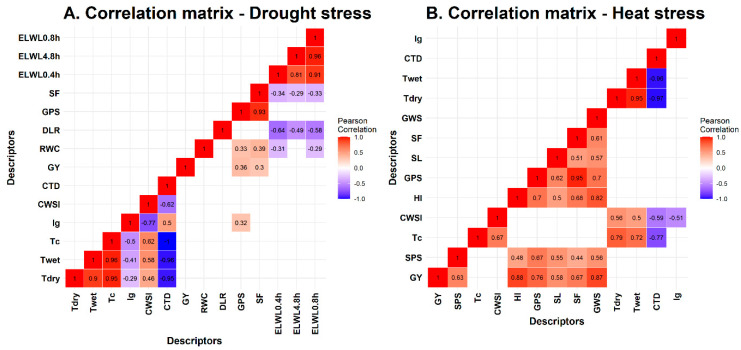
Correlation matrix plots for the drought and heat stress related traits recorded for (**A**) drought and (**B**) heat stress experiments.

Correlation matrix plots describing the degree of correlations between recorded traits (*p* < 0.05) are shown in Figure 6. Under DS, T_dry_, T_wet_, and T_c_ showed a strong positive correlation among themselves. In contrast, CTD showed a strong negative correlation with T_dry_, T_wet_, and T_c_. Similarly, ELWL_0–4h_, ELWL_4–8h_, and ELWL_0–8h_ showed weak but significant correlation with spike fertility and moderate but significant correlation with days to leaf rolling. For HS, grain yield had a significant positive correlation with spikelets per spike, harvest index, grains per spike, spike length, spike fertility, and grains weight per spike. Results showed that CTD showed a strong negative correlation with T_dry_ and T_wet_ compared than CWSI and T_c_.

**Figure 7:**
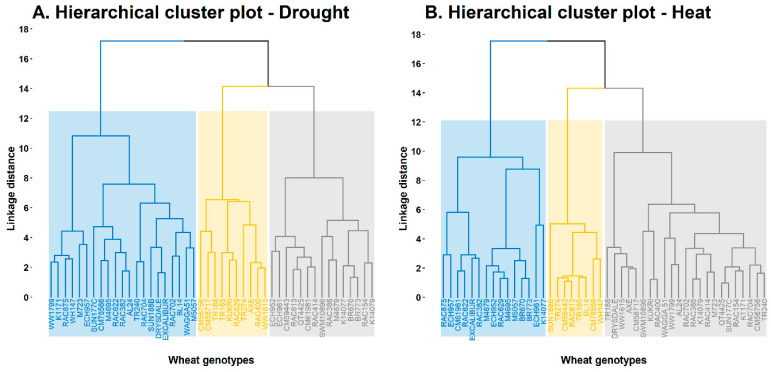
Hierarchical cluster analysis based on the similarity and dissimilarity of genotypes under (**A**) drought and (**B**) heat stress. The *x*-axis represents the genotypes, and the *y*-axis denotes the linkage distance of cluster pairs, where a higher value indicates a greater dissimilarity.

A cluster analysis was also performed, and genotypes were grouped based on their performance under DS and HS (Figure 7). Each cluster was further segmented into clades and leaf nodes, representing genotypes at the bottom. The arrangement and height of leaf nodes within each clade explain the degree of similarity or dissimilarity among genotypes—the genotypes grouped at the same leaf height performed similar and vice versa. Under DS, three clusters were observed, dissociated at a clade linkage distance of 17.19, 14.16, and 14.16, respectively. Cluster one grouped 21 genotypes; cluster two grouped ten genotypes; and cluster three grouped 15 genotypes at a clade linkage distance of 10.82, 7.59, and 8.01, respectively. RAC875 and WH147 performed similarly under DS (grouped at the same leaf height) compared with Drysdale and Excalibur, grouped at different leaf heights and clade within the same cluster (Figure 7).

For HS, three clusters were observed, dissociated at a clade linkage distance of 17.54, 14.30, and 14.30, respectively. Cluster one grouped 15 genotypes at a linkage distance of 9.57. Cluster two grouped eight genotypes at a linkage distance of 6.37, and cluster three grouped 23 genotypes at a linkage distance of 9.91. RAC875 and ECH957 performed similarly under HS (grouped at the same leaf height) compared with ECH961 and K14077, which were grouped at different leaf heights and clade within the same cluster (Figure 7).

## 4. Discussion

Germplasm screening for drought and heat stress in a controlled environment is an effective strategy for selecting plants with desirable traits for future research [23]. Early vigour is a complex trait influenced by many factors for its phenotypic expression in a Mediterranean environment where intermittent and low rainfall prevails. The rate of early leaf area development with their phyllochron interval determines maximum early root development and higher nitrogen uptake [31,48]. In this study, Mexican wheat genotypes produced a smaller leaf area than most Australian-origin genotypes (Table 1). This may be due to smaller phyllochron intervals, as longer leaves take more time to develop. This finding contradicts Rebetzke and Richards [31]’s results suggesting that Australian wheat cultivars are less vigorous in their early growth stage and exhibit smaller leaf area development. The present study showed the contribution of early vigour to the final grain yield, but due to complex post-anthesis genotype × environment interaction, the average grain yield benefit from the higher early vigour was not significant. These findings are consistent with Wilson et al. [49] and Zhao el al. [50], who stated that the translation of early vigour to higher grain yield was associated with uniform soil moisture availability throughout the plant’s life cycle. In the current study, genotypes also showed significant variation for yield components. These findings agree with Mahrookashani el al. [51] that spike fertility, harvest index, grains number per spike, and grain yield are susceptible to drought and heat stress. Plant attributes like above-ground dry matter or harvest index are less influenced by terminal DS and HS as these are mainly determined by the plants in their vegetative phase, which was finished when the DS and HS treatment started.

The ANOVA (Table 2) and genetic variability analysis (Table 3) indicate that significant variation existed for all the recorded traits for the drought and heat stress study. Blocking had substantial effects on treatment and all the measured traits. Previously, Deshmukh et al. [52] classified PCV and GCV into low (<10%), medium (10–20%), and high (>20%) categories (Figure 3). In the current study, grain yield and associated yield components had medium to high PCV and GCV for all the traits, as reported by Ogunniyan and Olakojo [53]. However, the δ^2^p classification into δ^2^g and δ^2^e is not enough for complete information on any source material. Like the coefficient of variation, genetic advance over mean can also be classified into low (<10%), medium (10–20%), and high (>20%) categories [54]. Higher estimates of GA and GAM confirmed the role of genetic effects on recorded traits, suggesting that simple genotypic selection is enough to improve targeted traits [55]. It also indicates that due to higher calculated GAM in most of the traits and inherent genetic diversity of experimental genotypes, the panel of 46 genotypes proved to be a valuable genetic resource for further drought and heat stress studies. The analysis of variance for yield and yield components provides valuable information concerning dominance, additive effects, and interaction [56]. This means adequate variability was present to distinguish groups of genotypes under DS and HS. The low CV of the recorded traits might be due to homogeneity within each block. The total variation presented in the targeted traits indicates the distinctiveness of genotypes from each other.

Different yield components have also been used to screen genotypes for drought and heat stress. Severe DS and HS tended to accelerate plant growth and senescence earlier, resulting in lower grains weight per spike due to the insufficient mobilization of metabolites and shorter grain filling duration [57,58]. The flag leaf senescence and chlorophyll fluorescence could measure the genotypic expression of the stay-green trait of the wheat genotypes under DS and HS. The genotypes like Excalibur, M4679, M5057, RAC386, RAC875, SUN188B, and TR274 are more consistent in maintaining their stay-green phenotype, which has a beneficial role in terminal DS and HS adaptation [59]. The stay-green phenotype is indicative of plant health and nitrogen status, which is closely linked to the amount of chlorophyll content [60]. Phenological responses like days to 50% anthesis and early maturity are important drought-escape mechanisms and key determinants for sustaining grain yield under DS [61]. These attributes enable them to efficiently utilize soil moisture during critical phenophases under stress [62]. However, it is also important that plant’s growth cycle should not be too short as this will compromise grain yield as evidenced by early maturing genotypes K14079 (D50A; 43.29) and K14077 (D50A; 44.29) having lower grain yield. Mwadzingeni et al. [23] also suggested that early maturing genotypes with lower grain yield might reflect an inefficiency in accumulating stem reserves. It is important to note that early maturing genotypes RAC875, WH147, Drysdale, and Excalibur excelled under DS with higher grain yield mainly due to higher grains weight per spike (Table 4). This might have resulted due to the stay-green trait of the genotypes and their extended grain-filling period. Higher grains weight per spike may be due to lower grain numbers per spike after HS and a proportionally greater availability of assimilates to the fewer kernels. This result agrees with Slafer et al. [63], suggesting a strong negative correlation between grains weight per spike and grains number per spike in wheat. A positive correlation of grain yield with yield components under both DS and HS suggests that grain yield could be enhanced by improving grains per spike and grains weight per spike (Figure 5A,B).

The evaluation of ELWL and RWC as screening techniques in drought stress has been associated with improved morpho-physiological and anatomical traits in wheat [64]. The usefulness of the cell membrane thermostability for the overall assessment of plant thermotolerance is well established. The RCI could be used as a useful index for drought and heat stress studies at an early growth stage in wheat [65]. Tolerant genotypes had higher cell membrane thermostability and less relative cell membrane injury. Our results also support these observations as higher grain yield genotypes had relatively less damage to the cell membrane, low ELWL, and higher RWC (Table 4 and Table 5).

Non-destructive infrared thermal imaging is an important technique to measure plant water status and canopy conductance based on T_c_ under DS or HS [21,37,38]. In the current study, significant differences for T_c_ were observed across the genotypes. Results showed that the use of T_c_ to assess the level of water stress on the genotypes depends upon stomatal conductance (Table 4 and Table 5). The closed stomata lead to energy dissipation, increasing T_c_ under DS and HS [66]. The genotypes like RAC875, Excalibur, RAC622, and ECH957 performed better under E1 and E2 by keeping their T_c_ at a metabolically suitable range for photosynthesis through canopy conductance.

As an index for irrigation scheduling, CWSI has been successfully used to study the differential response of the screening population in water stress studies. Results showed an inverse linear relationship between grain yield and CWSI, and it could be used as an important water stress index to evaluate plant water stress. However, CWSI fluctuates rapidly with the growing environment, which makes it difficult to choose the right value for each genotype to indicate real water stress [67]. Secondly, the response of the plant’s phenological stage to CWSI is unstable and fluctuates with the growing environment and genotypic response.

A canopy temperature depression measures the effectiveness of the canopy cooling capacity in maintaining the T_c_ under increasing atmospheric temperature. The canopy temperature depression has been recognized as a key attribute for characterizing genotypic responses to various environmental stresses [38,68]. In the current study, although T_a_ remained close to 25 °C for all the genotypes, a significant variation for CTD was observed across the genotypes both in E1 and E2. Under DS, the genotypes (RAC875, Drysdale, Excalibur, RAC704) with higher positive CTD values imply that they had managed T_c_ to a metabolically suitable level even under soil moisture deficit conditions. A cooled canopy ultimately leads to higher photosynthesis and more above-ground dry matter accumulation. This might be due to the effectiveness of the rooting system of responding genotypes in extracting soil moisture to maintain T_c_ [68].

Previously, a good association was found between I_g_ and g_s_ while studying the water status of different horticultural crops such as grapevines [37], olive orchards [69], and field crops such as wheat [66]. Although I_g_ is linearly related to g_s_, I_g_ is primarily attributed as a water stress indicator [70]. In the current experiment, a significant variation was observed for I_g_ values across the genotypes (Table 4 and Table 5), and this variation can be attributed to the (i) differences in the genotype’s response to g_s_ under DS or HS and (ii) varying water requirements of the genotypes due to differences in the developmental stage. Plants respond rapidly to regulate T_c_ in a changing plant water status by opening and closing stomata through leaf gas exchange fluxes. The modelling of I_g_ and CWSI shows that both indices have similar discriminative power. Due to a linear relationship with g_s_, I_g_ data recording is recommended, at least at the leaf level [70].

The principal component analysis is a dimensionality reduction multivariate technique that extracts the relevant information from the original data and allows the visualization of variables (loadings) and objects (scores) on the factor map [71]. In the current study, a large set of quantitative variables was categorized into different groups based on their homogeneity and dissimilarity. A relatively small proportion of the total explained variance for the E1 biplot indicates the inter-relationship complexity between the recorded traits. The PCA biplots for E1 and E2 show that IRTI, along with yield components (grain yield, spike length, spike fertility, harvest index, spikelets per spike, and grains per spike), had a higher contribution on the PCs and had a greater influence in the selection process (Table 6). These results further emphasize the importance of yield components in the selection process of genotypes [23]. Secondly, each genotype’s distance from the centre of gravity is defined based on the squared cosine (cos^2^), and it shows the importance of PCs for a given variable. The cos^2^ represents the contribution of PCs to the squared distance of the variable on the factor map. Therefore, the value of cos^2^ from the origin can help find PCs that are important to interpret both supplementary and active observations [71]. The vector on the factor map approximates the column information (phenotypic components), and the cos^2^ points approximate the row information (genotypes). Genotypes with a higher cos^2^ value on either side of the PCs indicate the quality representation of phenotypic components on the factor map [72]. The distance among the cos^2^ points shows the magnitude of similarity and dissimilarity between the corresponding genotypes. When two vectors have higher sharp angles with each other, the correlation between them is weak, and they are independent of each other. The same direction and smaller acute angles between dimension vectors indicate an inter-relationship among the trait variable concerning discriminating wheat genotypes.

## 5. Conclusions

The screening of wheat genotypes for terminal drought and heat stress is valuable for future research experiments that integrate drought or heat stress tolerance studies. Significant genotypic variation among IRTI water stress indices (T_c_, CTD, I_g_, T_dry_, T_wet_, and CWSI) and other physiological measures indicated that most of the traits expressed differentially among tolerant and susceptible genotypes under DS or HS conditions. This study reveals that with other morpho-physiological attributes, IRTI proved to be an important constructive technique for a spatially explicit assessment of plant water status under DS and HS. As a high-throughput phenotyping approach based on plant–water status, IRTI also provides a powerful insight to crop breeding programs for evaluating a large group of genotypes for their adaptability to drought or heat stress in a typical field condition. The stay-green trait provides valuable information for the diagnosis of plant photosynthesis status and to assess the health of the other plant physiological systems. Results from this study indicated that genotypes identified as drought and heat-tolerant (ECH957, RAC875, Excalibur, M723, Drysdale, RAC622) or susceptible (Kukri, CM59443, TR274, TR165) based on a set morpho-physiological measures including IRTI could potentially be used as a useful genetic resource in future drought and/or heat stress related pre-breeding and breeding programs. However, the performance of these selected genotypes requires further evaluation to assess their growth and yield performances in a natural field environment under drought or heat stress conditions.

## Figures and Tables

**Table 1:** List of wheat genotypes used in this study.

Wheat Genotypes	Origin	Type	Exp. Code
KUKRI	Australia	Cultivar	G1
DRYSDALE	Australia	Cultivar	G2
RAC875	Australia	Institute ID	G3
EXCALIBUR	Australia	Cultivar	G4
AXE	Australia	Cultivar	G5
CM56756	Mexico	Breeding line	G6
CM58717	Mexico	Breeding line	G7
SWM10896	Mexico	Breeding line	G8
CM61981	Mexico	Breeding line	G9
CM78566	Mexico	Breeding line	G10
CM59443	Mexico	Breeding line	G11
WH147	Mexico	Breeding line	G12
RAC154	Australia	Breeder designation	G13
RAC382	Australia	Breeder designation	G14
RAC386	Australia	Breeder designation	G15
RAC400	Australia	Breeder designation	G16
RAC414	Australia	Breeder designation	G17
M723	Australia	Breeder designation	G18
WAGGA51	Australia	Breeder designation	G19
BR670	Australia	Breeder designation	G20
BR773	Australia	Breeder designation	G21
TR165	Australia	Breeder designation	G22
TR188	Australia	Breeder designation	G23
ECH957	Australia	Breeder designation	G24
ECH952	Australia	Breeder designation	G25
ECH961	Australia	Breeder designation	G26
K14077	Australia	Breeder designation	G27
K14079	Australia	Breeder designation	G28
TR240	Australia	Breeder designation	G29
TR274	Australia	Breeder designation	G30
RAC702	Australia	Breeder designation	G31
RAC704	Australia	Breeder designation	G32
RAC613	Australia	Breeder designation	G33
RAC622	Australia	Breeder designation	G34
RAC629	Australia	Breeder designation	G35
WW1615	Australia	Breeder designation	G36
WW1799	Australia	Breeder designation	G37
QT4425	Australia	Breeder designation	G38
AL24	Australia	Breeder designation	G39
BL14	Australia	Breeder designation	G40
SUN177C	Australia	Breeder designation	G41
SUN188B	Australia	Breeder designation	G42
K1171	Australia	Breeder designation	G43
M4679	Australia	Breeder designation	G44
M4695	Australia	Breeder designation	G45
M5057	Australia	Breeder designation	G46

**Table 2:** Analysis of variance for grain yield and yield components of the 46 wheat genotypes grown under drought and heat-stressed environments. Mean square values and significance tests at 5% significance level for related traits are shown below.

	Source of Variation	df	D50A	GPS	GWS	GY	HI	SF	SL	SN	SPS
**Drought stress**	Block	3	128.81 **	16.38 **	0.05 **	1.80 **	59.87 *	130.69 **	0.23 ^ns^	16.37 **	1.64 ^ns^
	Genotypes (G)	45	50.28 **	47.18 **	0.05 **	1.59 **	81.45 **	495.01 **	1.83 **	13.83 **	18.64 **
	Check (C)	4	13.18 **	117.72 **	0.02 **	3.08 **	140.64 **	1382.50 **	2.35 **	1.32 *	48.39 **
	Test genotypes (G_t_) and G_t_ vs. C	41	53.90 **	40.30 **	0.05 **	1.45 **	75.68 **	408.43 **	1.78 **	15.05 **	15.74 **
	Residuals	12	0.53	0.54	0.00073	0.02	12.01	2.95	0.42	0.36	1.43
**Heat stress**	Block	3	-	50.82 **	0.04 **	2.62 **	30.09 ^ns^	509.03 **	1.09 **	14.23 *	5.75 *
	G	45	-	59.50 **	0.07 **	10.64 **	155.94 **	557.67 **	2.38 **	14.04 **	24.30 **
	C	4	-	67.09 **	0.10 **	19.62 **	412.42 **	541.97 **	1.84 **	21.57 **	38.86 **
	G_t_ and G_t_ vs. C	41	-	58.76 **	0.07 **	9.77 **	130.92 **	559.20 **	2.44 **	13.30 **	22.88 **
	Residuals	12	-	2.08	0.01	0.41	9.61	18.96	0.16	3.64	1.39

Days to 50% anthesis (D50A); grains per spike (GPS); grains weight per spike (GWS); grain yield (GY); harvest index (HI); spike fertility (SF); spike length (SL); spike number (SN); spikelets per spike (SPS). ^ns^ *p* > 0.05; * *p* < 0.05; ** *p* < 0.01; *p* < 0.001.

**Table 3:** Genetic variability analysis for various agronomic traits and yield components of the 46 wheat genotypes grown under drought and heat-stressed environments.

	Trait	PV	GV	EV	GCV	GCV Category	PCV	PCV Category	ECV	GA
Drought stress	GY	1.54	1.52	0.02	31.02	High	31.25	High	3.80	2.53
	SPS	16.14	14.71	1.43	14.39	Medium	15.07	Medium	4.48	7.55
	GPS	39.91	39.37	0.54	41.31	High	41.59	High	4.82	12.86
	HI	64.30	52.29	12.01	25.48	High	28.25	High	12.21	13.45
	PHT	148.32	148.03	0.29	24.10	High	24.13	High	1.07	25.08
	D50A	58.34	57.81	0.53	14.34	Medium	14.40	Medium	1.38	15.61
	SN	16.48	16.12	0.36	43.33	High	43.81	High	6.46	8.19
	SF	381.85	378.90	2.95	34.60	High	34.73	High	3.05	40.0
	SL	1.80	1.38	0.42	15.17	Medium	17.30	Medium	8.32	2.13
	GWS	0.06	0.06	0.00073	48.92	High	49.23	High	5.55	0.49
Heat stress	GY	7.54	7.13	0.41	52.09	High	53.57	High	12.5	5.36
	SPS	19.7	18.31	1.39	16.3	Medium	16.91	Medium	4.49	8.51
	GPS	43.77	41.68	2.08	44.07	High	45.15	High	9.85	13.0
	HI	110.27	100.66	9.61	39.02	High	40.84	High	12.06	19.78
	SN	14.26	10.61	3.64	22.27	High	25.80	High	13.04	5.80
	SF	450.78	431.82	18.96	38.27	High	39.10	High	8.02	41.96
	SL	2.37	2.21	0.16	19.51	Medium	20.21	High	5.30	2.96
	GWS	0.06	0.06	0.01	64.36	High	67.17	High	19.25	0.48

Abbreviations are: phenotypic variation (PV); genotypic variation (GV); environmental variation (EV); phenotypic coefficient of variance (PCV); environmental coefficient of variance (ECV); genotypic coefficient of variance (GCV); genetic advance (GA). Traits abbreviations: grain yield (GY); spikelets per spike (SPS); grains per spike (GPS); harvest index (HI); plant height (PHT); days to 50% anthesis (D50A); spike number per genotype (SN); spike fertility (SF); spike length (SL); grains weight per spike (GWS).

**Table 4:** Adjusted mean values of the ten best-performing and five low-performing genotypes under drought stress. Genotypes are ranked based on enlisted traits in the table.

Genotypes	GY	D50A	CTD	CWSI	DLR	ELWL	GPS	GWS	HI	I_g_	PHT	RWC	SF	SL	SN	SPS	T_c_	T_dry_	T_wet_
Top Performing Ten genotypes																			
ECH957	8.87	61.43	0.87	0.58	10.73	57.18	26.53	0.98	44.71	0.72	80.63	75.26	81.21	9.82	9.00	32.77	24.13	24.97	22.98
RAC875	5.60	47.73	1.62	0.47	10.02	52.81	23.17	0.52	39.63	1.11	51.83	78.77	82.23	8.79	10.50	28.17	23.28	24.33	22.52
M723	5.97	57.76	0.91	0.64	11.73	46.69	15.87	0.40	23.61	0.57	71.97	71.39	64.15	7.32	15.0	24.77	24.09	24.66	23.09
DRYSDALE	4.75	50.42	2.98	0.41	10.04	69.41	22.33	0.48	34.63	1.42	48.66	77.45	73.26	8.42	9.75	30.50	22.02	22.85	21.43
WH147	4.73	47.32	0.90	0.54	9.39	72.38	28.47	0.51	25.29	0.84	23.10	76.32	89.48	8.65	9.20	31.97	24.10	25.10	22.91
EXCALIBUR	4.30	51.50	2.75	0.41	10.08	56.81	18.50	0.45	41.09	1.42	51.50	76.59	84.09	7.42	9.50	22.00	22.25	23.21	21.57
RAC622	5.54	62.96	0.89	0.59	7.63	64.42	13.93	0.35	28.68	0.70	52.83	83.89	45.85	7.82	15.60	30.37	24.11	24.81	23.12
RAC704	4.22	47.29	2.88	0.43	6.29	78.69	11.93	0.36	40.18	1.31	44.16	74.34	43.17	7.32	11.60	27.70	22.12	22.93	21.50
AL24	4.21	51.63	1.65	0.59	8.93	66.42	11.73	0.59	43.12	0.70	51.77	76.76	40.09	7.22	7.20	29.57	23.35	24.03	22.40
BR670	4.20	58.43	0	0.67	11.39	45.77	15.87	0.35	34.33	0.50	60.63	81.19	69.91	7.82	12.0	22.77	26.64	27.50	24.92
Low performing five genotypes																			
KUKRI	3.25	48.62	0.54	0.74	6.94	79.46	13.67	0.35	26.78	0.35	55.75	70.06	48.87	6.88	9.25	28.0	24.46	25.02	22.85
ECH952	2.74	52.43	0	0.59	10.06	67.91	12.53	0.46	18.72	0.70	55.63	64.82	50.45	7.48	6.0	24.77	25.11	25.47	24.58
TR165	2.68	50.43	0	0.82	8.39	66.92	17.87	0.54	18.59	0.22	53.63	73.31	65.20	8.48	5.0	27.43	25.70	26.09	24.01
TR274	2.57	51.63	0	0.90	6.96	99.42	9.93	0.33	20.43	0.64	46.16	73.35	39.93	7.32	7.60	25.03	25.37	25.96	25.93
CM59443	2.55	49.65	0	0.75	9.39	80.22	8.47	0.49	23.81	0.34	42.10	81.47	29.42	7.98	5.20	28.63	26.51	27.05	24.91

Abbreviations are: grain yield (GY), days to 50% anthesis (D50A), chlorophyll fluorescence (F_v_/F_m_), canopy temperature depression (CTD), crop water stress index (CWSI), days to leaf rolling (DLR), excised leaf water loss after 0–8 hr of leaf excision (ELWL), grains per spike (GPS), grains weight per spike (GWS), harvest index (HI), infrared index (I_g_), plant height (PHT), relative water contents (RWC), spike fertility (SF), spike length (SL), spike number (SN), spikelets per spike (SPS), canopy temperature (T_c_), reference temperature of the stressed crop (T_dry_), reference temperature of the non-stressed crop (T_wet_).

**Table 5:** Adjusted mean values of the ten best-performing and five low-performing genotypes under heat stress. Genotypes are ranked based on enlisted traits in the table.

Treatment	GY	RCI	CTD	CWSI	GPS	GWS	HI	I_g_	SF	SL	SN	SPS	T_c_	T_dry_	T_wet_
Top performing ten genotypes															
ECH957	11.35	16.90	0	0.29	23.83	0.95	35.34	0.68	68.31	10.02	11.85	35.03	25.40	26.03	24.48
AXE	10.73	12.16	0	0.83	20.77	0.82	43.49	0.73	68.97	9.35	13.25	29.97	29.11	28.96	26.23
RAC875	9.90	16.08	0.92	0.28	27.17	0.61	43.96	0.76	88.98	8.50	16.25	30.50	25.20	24.66	23.32
TR188	10.40	10.68	0	0.65	23.83	1.30	51.97	0.49	88.03	8.85	7.85	27.03	28.16	29.13	27.93
DRYSDALE	10.03	12.70	0	0.64	28.77	0.55	46.43	0.60	95.94	8.85	18.25	29.97	28.06	28.79	27.81
EXCALIBUR	7.87	26.25	0.18	0.23	19.67	0.32	25.35	0.70	80.50	7.58	18.50	24.50	24.66	25.66	23.61
RAC622	9.43	5.58	0	0.20	16.77	0.51	31.90	1.10	52.16	7.65	18.05	31.43	25.22	26.08	25.45
TR240	7.60	11.41	0	0.79	14.10	0.49	34.84	0.96	50.21	8.32	15.02	27.43	29.00	29.57	28.56
CM61981	7.00	20.42	0	0.27	14.10	0.38	29.28	0.84	49.83	8.35	18.25	27.97	25.63	25.84	24.38
WAGGA51	6.50	20.93	0	0.28	15.17	0.50	26.30	1.52	61.12	8.35	12.85	25.03	25.75	29.47	28.38
Low performing five genotypes															
KUKRI	5.88	12.43	0	0.74	24.33	0.49	25.97	0.35	73.72	8.17	12.25	33.00	28.73	28.90	26.74
CM59443	2.74	12.23	0	0.78	7.43	0.25	16.62	0.43	31.78	7.35	11.25	22.63	28.72	29.01	27.37
TR274	1.90	20.27	0	0.66	4.77	0.09	15.16	0.65	15.65	7.15	18.05	26.10	28.61	29.18	27.80
CM78566	1.16	14.80	0	0.79	2.77	0.07	7.36	0.35	15.33	6.35	15.25	15.97	28.54	28.14	25.68
WH147	1.16	18.81	0	0.67	3.43	0.06	9.34	0.56	18.80	3.52	18.25	16.63	28.97	28.85	27.79

Abbreviations are: grain yield (GY), relative cell membrane injury (RCI), canopy temperature depression (CTD), crop water stress index (CWSI), grains per spike (GPS), grains weight per spike (GWS), harvest index (HI), infrared index (I_g_), spike fertility (SF), spike length (SL), spike number (SN), spikelets per spike (SPS), canopy temperature (T_c_), reference temperature of the stressed crop (T_dry_), reference temperature of the non-stressed crop (T_wet_).

**Table 6:** Rotated principal component matrix based on the most contributing ten traits for drought and heat stress experiments.

	Drought Stress			Heat Stress	
Traits	PC-1	PC-2	Traits	PC-1	PC-2
Tdry	0.883	0.181	GY	0.899	0.218
Twet	0.889	0.288	SPS	0.675	0.282
Tc	0.944	0.266	Tc	−0.298	0.832
Ig	−0.522	−0.413	CWSI	−0.382	0.644
CSWI	0.568	0.576	HI	0.838	0.237
CTD	−0.944	−0.266	GPS	0.907	0.164
GY	−0.061	−0.341	SL	0.664	0.312
RWC	0.272	−0.419	SF	0.839	0.073
DLR	0.446	−0.530	GWS	0.834	0.284
GPS	0.074	−0.634	Tdry	−0.267	0.915
Explained variance (eigenvalue)	4.883	3.766	Explained variance (eigenvalue)	5.084	4.029
Proportion of total variance (%)	34.879	26.903	Proportion of total variance (%)	39.110	30.989
Cumulative percent of variance (%)	34.879	61.782	Cumulative percent of variance (%)	39.110	70.098

## Data Availability

The data is contained within the article.
